# Effects of Probiotics on Growth, Survival, Water Quality and Disease Resistance of Red Hybrid Tilapia (*Oreochromis* spp.) Fingerlings in a Biofloc System

**DOI:** 10.3390/ani11123514

**Published:** 2021-12-09

**Authors:** Aimi Zabidi, Fatimah Md Yusoff, Nurul Amin, Nur Jasmin Mohd Yaminudin, Puvaneswari Puvanasundram, Murni Marlina Abd Karim

**Affiliations:** 1 Department of Aquaculture, Faculty of Agriculture, Universiti Putra Malaysia, Serdang 43400, Malaysia; nuraimizabidi@yahoo.com (A.Z.); fatimahyus@gmail.com (F.M.Y.); smnabd@gmail.com (N.A.); jasumin91@gmail.com (N.J.M.Y.); 2 Laboratory of Sustainable Aquaculture, International Institute of Aquaculture and Aquatic Sciences, Universiti Putra Malaysia, Port Dickson 71050, Malaysia; 3 Laboratory of Aquatic Animal Health and Therapeutics, Institute of Biosciences, Universiti Putra Malaysia, Serdang 43400, Malaysia; puvaneswari.p.s@gmail.com

**Keywords:** biofloc streptococcus agalactiae, red hybrid tilapia, disease resistance, survival

## Abstract

**Simple Summary:**

Streptococcosis, a warm-water pathogenic bacteria, has greatly affected red hybrid tilapia production in Malaysia over the years, causing mass mortality in various culture systems. Probiotics have been used to treat and prevent bacterial diseases, including streptococcosis, yet they require constant application to ensure that their concentration is adequate. Incorporating probiotics in a biofloc system may reduce this issue as the effective microorganism may further flourish and be utilized by the fish. The objectives of this study were to evaluate the influence of probiotic addition on the growth performance and water quality of red hybrid tilapia. From the study, it was observed that a probiotic mix was able to inhibit *Streptococcus* spp., improve red hybrid tilapia performance and disease resistance against streptococcosis. Providing a beneficial mix of probiotics can effectively improve biofloc culture in red hybrid tilapia culture.

**Abstract:**

Biofloc technology has shown positive effects in aquaculture, especially on the growth performance of cultured animals. The aims of this study were to evaluate the effects of adding different probiotic strains in a biofloc system on the growth performance and disease resistance of red hybrid tilapia (*Oreochromis* spp.). Three different probiotics (*Lysinibacillus fusiformis* SPS11, *Bacillus amyloliquefaciens* L9, and *Enterococcus hirae* LAB3), commercial probiotics (MG1) and a mixed probiotics (MP) combining all three strains were used in this study. The in vitro assay results showed that the mixed probiotic (MP) was able to inhibit the growth of *Streptococcus agalactiae* and *Streptococcus iniae* significantly compared to the single and commercial probiotic. The efficacy of MP was further tested in in vivo tilapia culture challenged with *S. agalactiae.* The best specific growth rate (3.73 ± 0.23% day^−1^) and feed conversion ratio (0.76 ± 0.04) were recorded in the group of biofloc with addition of MP. After being challenged with *S. agalactiae*, the group of biofloc with MP had significantly higher survival (83 ± 1.43%) compared to the other groups. Furthermore, the nitrogen concentration (NO_2_-N and NH_4_-N) was significantly lower in all the biofloc groups compared to the control. Hence, the addition of probiotics was able to provide beneficial effects to red hybrid tilapia culture in the biofloc system.

## 1. Introduction

Tilapia, *Oreochromis* sp., is a highly resistant species that can adapt to a wide range of environmental conditions; thus, little environmental modification, with a low-technology system, is needed for culturing it [1]. Tilapia has a high economical yield and has been cultured intensively throughout Asia, with China having the highest production, of roughly 1.7 million tonnes per year [2].

Tilapia is also the most cultured freshwater species in Malaysia due to its fast growth in tropical climates. In 2019, Malaysia produced up to 31, 884 metric tonnes of red hybrid tilapia, which contributed to 30% of Malaysia’s total aquaculture production [3]. Red hybrid tilapia, *Oreochromis* spp., is commonly cultured in cages located in former mining pools, rivers, irrigation canals, and lakes or reservoirs using either the semi-intensive or intensive methods [4].

Tilapia is considered more resistant to bacterial, parasitic, fungal, and viral diseases compared to other species of cultured fish. However, due to intensive culture practices, tilapia is becoming more susceptible to various diseases, mainly streptococcal infection caused by *Streptococcus* sp. [5]. *Streptococcus* sp. is known to cause the most significant bacterial diseases in Nile Tilapia, *Oreochromis niloticus* causing mass mortality and major economic losses [6].

In tilapia farming, streptococcal infections have become a major problem, resulting in severe financial losses [7,8]. *Streptococcus iniae* and *Streptococcus agalactiae* are the two most common bacteria that affect tilapia production around the world [9,10]. Streptococcal infection is a common bacterial disease in farm fish such as tilapia, with symptoms such as small, isolated lesions near the fish’s lip or tail line. Septicemia, hemorrhage, swirling behavior, bent bodies, anorexia, lethargy, and death are all symptoms of untreated infections, and they usually appear within seven days of the first symptoms [11].

The application of probiotics is a promising alternative to antibiotics for disease management in aquaculture. Probiotics are live microorganisms, which when administered in sufficient amounts, confer beneficial effects on the host [12]. The efficacy of probiotics is not just restricted to the gastrointestinal tract, but it plays a significant role in improving the overall health of the host by promoting growth, enhancing immune parameters, and improving the quality of the culture water [13]. The addition of probiotics to the aquaculture system has been proven to improve growth performance, water quality, and disease resistance of tilapia, *Oreochromis* sp. [14,15,16].

However, the continuous addition of probiotics to an intensive culture system may be difficult to maintain. Thus, biofloc technology (BFT) is a well recommended system through which to maintain efficient bacterial load, which includes beneficial bacteria that both prevent bacterial diseases and improve the growth performance of cultured fish in intensive culture systems, especially in tilapia cultures [17]. Enhanced immunity is commonly observed in BFT, which aids in protection against diseases while providing nonspecific defence against various pathogens [18].

Exogenous probiotic addition in a biofloc system may enhance the efficiency of probiotics as they are cultured in a closed system and are easily utilized by the fish in the system. The large microbial load in the biofloc system may function as a biological control agent against pathogens through competitive exclusion [19]. Incorporating beneficial probiotics in a biofloc system and producing quality floc may improve cultured organisms and eradicate bacterial disease as a whole. Selecting and testing suitable probiotic combinations that inhibit certain diseases are the most effective prevention measure for disease management in the culture system. Thus, the aims of this study were to evaluate the effects of adding probiotics on growth performance, survival, water quality, and disease resistance of red hybrid tilapia fingerlings in a biofloc system.

## 2. Materials and Methods

### 2.1. Preparation of Selected Probiotic

The probiotics used in this study were obtained from the Laboratory of Fish Health, Department of Aquaculture, Faculty of Agriculture, Universiti Putra Malaysia (UPM). *Lysinibacillus fusiformis* (SPS11) was previously isolated from microalgae [20], *Bacillus amyloliquefaciens* (L9) from blue swimming crab, *Portunus pelagicus* [21] and *Enterococcus hirae* (LAB3) from barramundi, *Lates calcarifer* [22]. A commercial probiotic (MG1) that consists of the probiotic strains *Lactobacillus* sp., *Aztobacter* sp. and *Azospirillum* sp. was used as the positive control.

All the probiotics were cultured on Trypticase Soy Agar (TSA: Difco™, Wayne, PA, USA) and incubated at 28 °C for 24 h. A pure culture of each probiotic was cryopreserved in replicates at −80 °C in a REVCO Ultra-Low freeze using suitable broth medium. A 20% glycerol stock was prepared using glycerol solution and autoclaved for 15 min at 121 °C. Bacteria samples from Trypticase Soy Broth (TSB: Difco™, USA) were mixed with the prepared glycerol solution at a 1:1 ratio and stored at −80 °C as long-term storage and −20 °C as working storage.

### 2.2. Pathogen Culture

Two strains of pathogen (*Streptococcus agalactiae* and *Streptococcus iniae*) used in this study were obtained from the Immunology Laboratory, Department of Aquaculture, Faculty of Agriculture, UPM. These pathogens were cultured on TSA and incubated at 30 °C for 24 h. All the selected probiotics were screened against these two pathogens to observe for their antagonistic properties.

### 2.3. In Vitro Screening of Probiotics

Both the pathogens and the probiotics were cultured in 10 mL of TSB and incubated overnight at 30 °C with shaking prior to use in the in vitro assay.

#### Well Diffusion Assay

The well diffusion method was used to test the ability of the probiotics to inhibit two pathogens, *S. agalactiae* and *S. iniae*. The concentrations of overnight culture of the single-strain probiotic (SPS11, L9, and LAB3) and MP were adjusted to 10^7^ CFU mL^−1^ while the concentrations of the pathogens (*S. agalactiae* and *S. iniae*) were adjusted to 10^6^ and 10^8^ CFU mL^−1^. The pathogen culture was then swabbed onto TSA agar plates using a sterile cotton swab. In the same agar, circular holes (wells) with a diameter of 5 mm were punched out and filled with 10 µL of probiotic culture (single and mixed) respectively. The plates were allowed to dry for 30 min and incubated at 28 °C for 18–24 h. Each group was performed in triplicates. The next day, the presence of clear inhibition zones was observed, measured, and recorded.

### 2.4. Addition of Probiotics in a Biofloc Culture System

#### 2.4.1. Red Hybrid Tilapia Fingerlings and Monitoring

The red hybrid tilapia fingerlings, *Oreochromis* spp. were obtained from the hatchery in UPM. The fingerlings were acclimatized for a week under laboratory conditions. The average body weight was 570 ± 3.5 mg with average length of 4.2 ± 0.8 cm. During acclimatization, the fish were fed twice daily with a commercial fish diet (Sea Master, PG-01), which contained 44% protein with 7% lipid (5% body weight) and maintained on illumination at 12 h/day at a constant temperature of 27–28 °C.

#### 2.4.2. Biofloc Preparation, Maintenance, and Experimental Trial

The experiment was carried out using 12 aquaria (capacity: 6 L each) at the UPM Research Station in Puchong for a duration of 4 weeks. Each aquarium was filled with 5 L of filtered freshwater treated with a chlorine solution (10 ppm). Illumination was provided for 12 h/day and the temperature maintained at a range of 27.3–28.3 °C throughout the experiment. Each tank was continuously aerated using air blowers to maintain the dissolved oxygen (DO) level of > 6 mg L^−1^ due to the high oxygen consumption and mixing intensity required in the biofloc tank. The fish fingerlings were distributed randomly in each group tank at a density of 20 fingerlings/tank (4 fingerlings/L). The groups included C (Control), B1 (Biofloc Control), MG1 (Biofloc with MG1), and MP (Biofloc with MP) (Table 1). All the groups were set up in triplicates. For the biofloc groups with probiotics, MG1 and MP were inoculated at the concentration of 5 × 10^8^ CFU mL^−1^.

Molasses was added to all the biofloc tanks at a ratio of 20:1 (C:N), using 20 g of carbon to transform 1 g of total ammonia nitrogen until the ammonia nitrogen was less than 1 mg L^−1^ [23,24]. The biofloc group tanks were filled with 5 L biofloc water obtained from a matured red hybrid tilapia biofloc tank (1 m^3^), which was maintained for 1 month prior to the experiment [17]. Additional freshwater was added for evaporation loss when the water fell below the 5 L mark and floc removal was performed on a weekly basis. Meanwhile, a 50% water change was performed every 3 days in the control tanks.

#### 2.4.3. Water Quality Monitoring

The water temperature, dissolved oxygen (DO), pH, and salinity were measured daily in situ in the morning and afternoon using a multiparameter (YSI model 556 MPS). The dissolved inorganic nitrogen (ammonia, nitrite, and nitrate) was measured twice a week using a multiparameter photometer (HANNA, HI 33300) following the procedures of the standard methods [25]. Biofloc volume (mL L^−1^) was determined by using the Imhoff cone once a week [26,27].

#### 2.4.4. Growth Performance

Every week, five fish were selected randomly from each group and sedated using MS222 at 20 mg L^−1^ buffered with two parts sodium bicarbonate prepared in a 4 L aquarium. Once the fingerling was slightly sedated, each fish was brought out to measure its weight (g) using a laboratory scale. Once measured, each fish was transferred into a clean freshwater tank with aeration provided to recover from the anaesthesia and moved back into its respective group. The growth performance of the tilapia fingerlings was calculated using the following equations.

$$\mathrm{Weight}\;\mathrm{gain}\;(\%)=\frac{mean\;final\;weight-mean\;initial\;weight\;}{mean\;initial\;weight}\times 100$$

$$\mathrm{Specific}\;\mathrm{growth}\;\mathrm{rate}\;(\%\;d^{-1})=\frac{mean\;final\;weight-mean\;initial\;weight}{Number\;of\;culture\;days}\times 100$$

Total feed intake (g) = Total amount of consumed food (g) in each group for 4 weeks.

Biomass gain (g) = Harvested biomass (g) − Stocked biomass (g)

$$\mathrm{Feed}\;\mathrm{conversion}\;\mathrm{ratio}=\frac{Total\;feed\;intake\;\left(g\right)}{Wet\;weight\;gain\;\left(g\right)}$$

$$\mathrm{Survival}\;(\%)=\frac{Number\;of\;harvested\;fish}{Number\;of\;stocked\;fish}\times 100$$

### 2.5. Challenge Test

After 4 weeks of the biofloc trial, thee remaining tilapia were used in the challenge assay against the pathogen *S. agalactiae*. *Streptococcus agalactiae* was used in the in vivo assay due to the ability of the mixed probiotics to inhibit the growth of this pathogen better than *S. iniae*. There were five groups: C, S1, S2, S3, and S4, as described in Table 2, and each group was set up in triplicates. After 4 weeks, a total of 10 fish from the respective groups were placed into a new aquarium filled with 2 L of dechlorinated freshwater. *Streptococcus agalactiae* was added after 24 h to each group at a concentration of 10^8^ CFU mL^−1^. The mortality and clinical signs of infection were recorded daily. The challenge test was ended when the control group challenged with *S. agalactiae* with no addition of probiotics reached 50% mortality or after 14 days.

### 2.6. Histopathological Examination

Three moribund fish were chosen randomly from each challenged group including three healthy fish from the CS group and euthanized with MS-222 (60 mg/L) buffered with two parts sodium bicarbonate before dissection. Next, selected organs such as the gills, liver, and kidney were dissected and promptly fixed in 10% (*v/v*) buffered formalin for histopathological inspection. The tissue samples were then sent for fixation at the Veterinary Laboratory Service Unit, Faculty of Veterinary, UPM. Samples were infiltrated, embedded in paraffin wax, and sectioned at 5 mm on a rotary microtome after being dehydrated in an ethanol series. The hematoxylin and eosin-stained sections were studied under a compound microscope (Zeiss Primo Star, Germany) for histopathological comparisons.

### 2.7. Statistical Analysis

All the data collected were run under a normality test and analyzed using one-way analysis of variance (ANOVA). Tukey’s post hoc test (*p* = 0.05) was used (IBM SPSS Statistic 20 software) to identify significant differences among all the groups. Results were expressed as the mean ± standard error and the difference was considered significant at *p* < 0.05.

## 3. Results

### 3.1. Probiotic Selection

**Figure 1:**
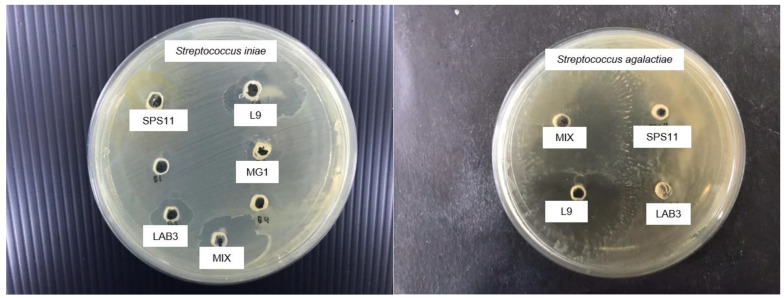
The inhibition zones demonstrated by probiotic *Lysinibacillus fusiformis* SPS11, *Bacillus amyloliquefaciens* L9, *Enterococcus hirae* LAB3, MP, and MG1 against *Streptococcus iniae* and SPS11, L9, LAB3, and MP against *Streptococcus agalactiae*.

Inhibition zones were observed in each probiotic group against *S. agalactiae* and *S. iniae* (Figure 1). All the single probiotics showed positive inhibition towards both pathogens (Table 3). Moreover, MP also exhibited inhibition zones at 10.2 ± 0.2 mm and 10.75 ± 1 mm against *S. agalactiae* and *S. iniae*, respectively.

### 3.2. Growth and S3.2 Growth and Survival

The growth performances of the red hybrid tilapia in the biofloc system with probiotics are presented in Table 4. Final body weight was significantly higher in the MP group which was recorded at 15.07 ± 1.30 g compared to the other groups. There was no significant difference on the survival among all the groups (>89%). The SGR and FCR were significantly higher (*p* > 0.05) in B1, MG1 and MP compared to the control. However, the best SGR (3.73 ± 0.23% day^−1^) and FCR (0.76 ± 0.04) were observed in the MP group.

### 3.3. Water Quality Parameters

The water quality parameters during this study are presented in Table 5. The water quality parameters of all the groups were within the optimum range. Temperature, DO and PH did not significantly differ (*p* > 0.05) among all the groups. Nevertheless, the nitrite (NO_2_-N) and ammonia (NH_4_-N) concentrations were significantly (*p* < 0.05) lower in all the biofloc groups while the control exhibited the lowest nitrate (NO_3_-N) concentration.

### 3.4. Challenge Assay of Red Hybrid Tilapia

**Figure 2:**
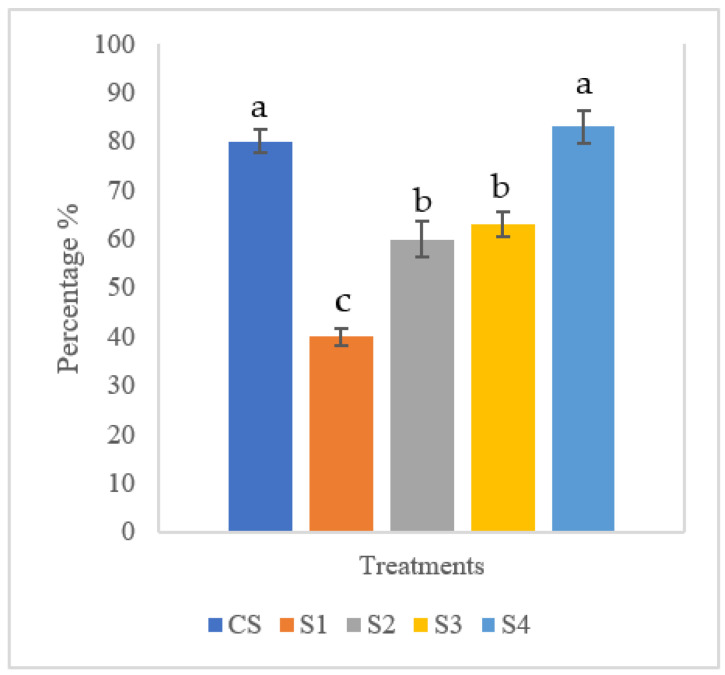
Survival (%) of tilapia in control (CS), S1 (*Streptococcus agalactiae*), S2 (Biofloc + *Streptococcus agalactiae*), S3 (Biofloc + MG1 + *Streptococcus agalactiae*), S4 (Biofloc + MP + *Streptococcus agalactiae*) challenged against *Streptococcus agalactiae.* Each value is the mean ± SEM of triplicate analysis. Superscript ^a,b,c^ on bars and mean with different alphabetical letters indicate a statistically significant difference (One-way ANOVA, *p* < 0.05).

The survival of the tilapia after 5 days of challenge from *S. agalactiae* is shown in Figure 2. The highest survival was recorded at 83 ± 1.43% in S4 (biofloc + MP + *Streptococcus agalactiae*), which was not significantly different (*p* > 0.05) from the control group (CS, 80 ± 0.82%). The fish in the S4 group showed significantly higher survival when compared to the group with *S. agalactiae* only (S1, 40 ± 0.34%).

### 3.5. Clinical Signs and Symptoms

**Figure 3:**
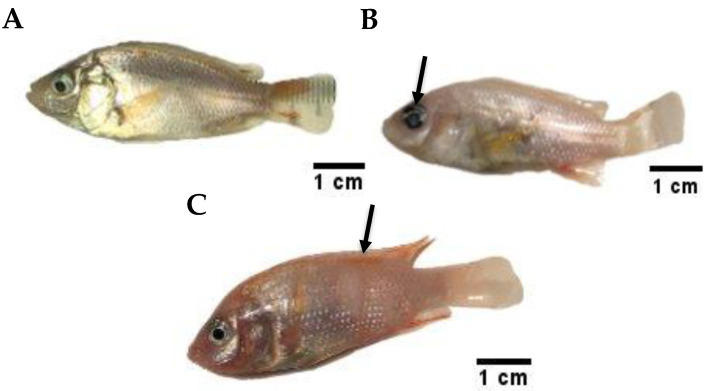
Clinical observation of red hybrid tilapia after challenge with *Streptococcus agalactiae*. A healthy fish in the control (**A**) and infected fish (**B**,**C**). Challenged fish showed eye opacity, redness (**B**) and lesions on the body (**C**) post-infection.

A normal, healthy red hybrid tilapia exhibits a laterally compressed to oval body shape, healthy, transparent and spherical eye lenses, as well as normal glistening skin with no pathological lesions or hemorrhage (Figure 3A). External eye redness or opacity and detached scales with signs of haemorrhage beneath the skin (Figure 3B) were observed in the infected fish. Skin lesions and ulcers were also observed in specimens following signs of stress: lethargy, passivity, and anorexia (Figure 3C).

### 3.6. Histopathological Examination

**Figure 4:**
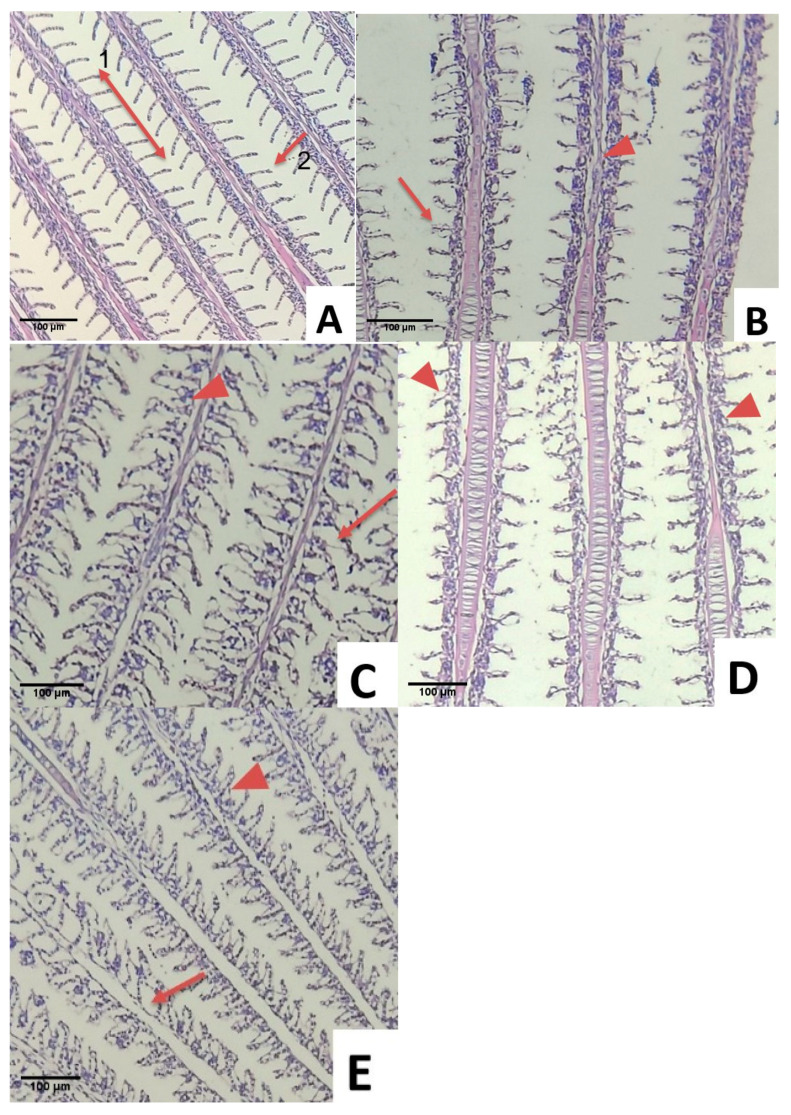
Histological changes in the gills of fish infected with *Streptococcus agalactiae* (**A**). Normal gill filaments in C (1: secondary lamella, 2: filament epithelium), (**B**). Vacuolation of interstitial space edema (thin arrow) and lamellar dilation (short arrow) in S1 (*Streptococcus agalactiae*), (**C**). Vacuolation of interstitial space edema (thin arrow) and lamellar dilation (short arrow) in S2 (Biofloc + *Streptococcus agalactiae*). (**D**). Aneurism on lamella (short arrow) in S3 (Biofloc + MG1 + *Streptococcus agalactiae*), (**E**). Aneurism (Short arrow) and lifting of respiratory epithelium (thin arrow) in S4 (Biofloc + MP + *Streptococcus agalactiae*). (HE staining, 100× magnification).

All the experimentally infected fish challenged with *S. agalactiae* exhibited multiple histopathological changes in the gill, kidney, and liver. Histopathological changes in the gills of the infected fish are presented in Figure 4A–E. The fish in CS showed a healthy gill with clear gill filaments including secondary lamella and uncongested filament epithelium (Figure 4A). Vacuolation of interstitial space edema and lamellar dilation were observed in the S1 group due to the agglomeration of red blood cells (aneurism). The complete fusion of several lamellae was also observed in S1 (Figure 4B). Multiple lamella vacuolation and dilation were observed in group S2 (Figure 4E). Respiratory distress was observed due to damage of gills in group S3 (Figure 4D). Minor aneurism and lifting of respiratory epithelium on multiple lamellas were found in S4 (Figure 4C).

**Figure 5:**
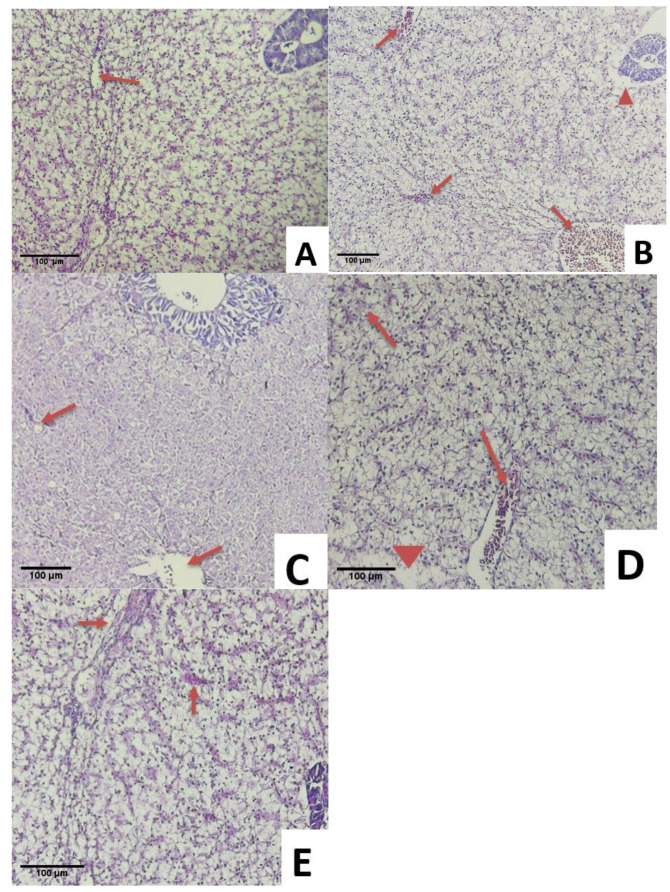
Histological changes in the liver of fish infected with *Streptococcus agalactiae*: (**A**). Vessel not congested (thin arrow) in CS, (**B**). Severely congested blood vessel (thin arrow) and cell necrosis (short arrow) in S1 (*Streptococcus agalactiae*), (**C**). Severely congested blood vessel (thin arrow) in S2 (Biofloc + *Streptococcus agalactiae*), (**D**). Congested blood vessel (thin arrow) and slight cell necrosis (short arrow) in S3 (Biofloc + MG1 + *Streptococcus agalactiae*), (**E**). Vessel not congested (thin arrow) in S4 (Biofloc + MP + *Streptococcus agalactiae*). (HE staining, 100× magnification).

Although uncongested blood vessels were observed in the livers of the fish in the CS group, there was no sign of swelling or necrosis (Figure 5A). Acute hepatic cellular swelling, nuclear pyknosis and blood vessel congestion were found in the fish of the S1 group (Figure 5B). Multiple congested blood vessels showed the onset of bacterial infection in group S2 (Figure 5C). Similarly, the main blood vessels in the liver were congested and followed by cell necrosis in S3 (Figure 5D). There was no vessel congestion in group S4, although eosinophilic infiltration was noticed in the liver tissues (Figure 5E). Overall, congestion and thrombosis of portal blood vessels in the liver tissues of the infected fish were revealed.

**Figure 6:**
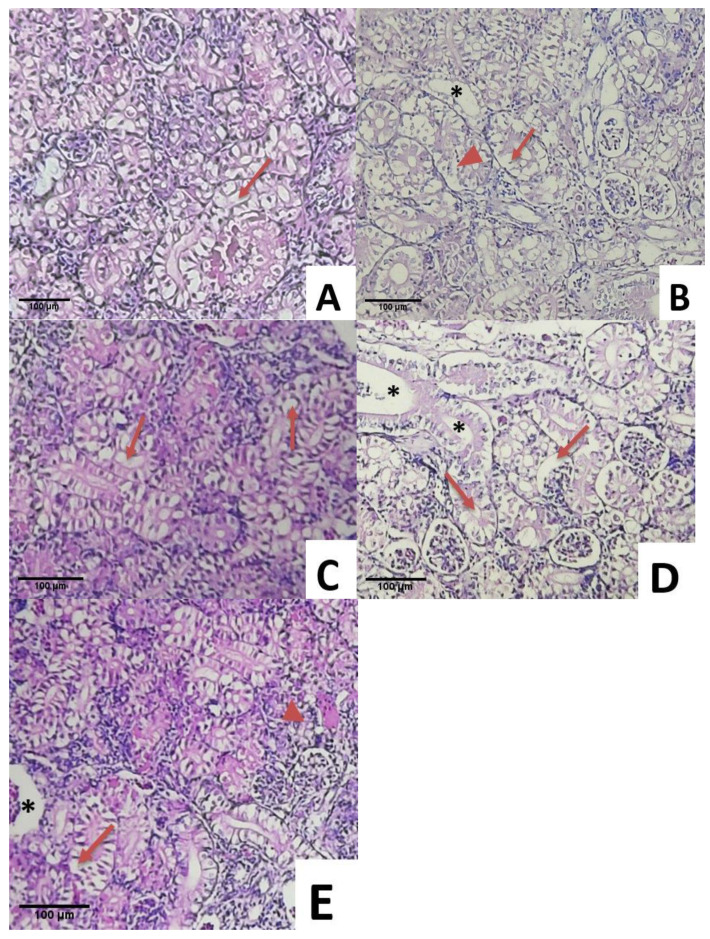
Histological changes in the kidneys of fish infected with *Streptococcus agalactiae*: (**A**).: Vacuolation (thin arrow) in CS, (**B**). Major vacuolation (thin arrow) necrosis and pyknotic nuclei (short arrow) and tubule necrosis (asterix) in S1 (*Streptococcus agalactiae*), (**C**). Vacuolation (thin arrow), pyknotic nuclei (short arrow) and tubule necrosis (asterix) in S2 (Biofloc + *Streptococcus agalactiae*), (**D**). Major vacuolation (thin arrow) tubule necrosis (asterix) in S3 (Biofloc + MG1 + *Streptococcus agalactiae*), (**E**). Slight vacuolation (thin arrow) in S4 (Biofloc + MP + *Streptococcus agalactiae*) (HE staining, 100× magnification).

Slight glomerular vacuolation was observed in the kidney samples of CS without *S. agalactiae* infection (Figure 6A). Renal alterations were observed in the fish samples in S1, which were characterized by haemorrhage and thrombosis in glomeruli and tubules as well as the infiltration of inflammatory cells and atrophy in hematopoietic tissues (Figure 6B). Similar glomerular vacuolation, pyknotic nuclei, and tubule necrosis were found in S2 (Figure 6C). Major glomerulus vacuolation and necrosis were detected throughout the kidney samples in the S3 group (Figure 6D). The kidney was overall intact with slight glomerular vacuolation in S4 (Figure 6E). It could be concluded that less tissue damage was observed in group S4.

## 4. Discussion

### 4.1. Probiotic Selection

In this study, the MP displayed inhibition zones against both *S. agalactiae* and *S. iniae* in the in vitro well diffusion assay. The clear inhibition zones displayed by the MP might have been due to the production of inhibitory compounds that caused competitive exclusion between probiotic and pathogen which was an important factor for good probiotic candidates [28]. All the single-strain probiotics used in this study had shown antagonistic activities towards other pathogens, mainly from the genus of *Vibrio*, in previous studies [20,21,22]. The results demonstrated significant improvements in the antagonistic properties by the MP against pathogenic *Streptococcus* sp. compared to the control group. This indicated the potential applicability of the MP in controlling diseases caused by pathogenic *Streptococcus* sp.

### 4.2. Growth Performance

The results of the present study demonstrated that the biofloc with mixed probiotic addition had significant effects on the growth performance of red hybrid tilapia fingerlings, which were similar to those shown by other studies [29,30]. The growth performance of the biofloc-treated group was much better than the CS group. The highest final body weight was observed in MP (15.07±1.30 g) as compared to the two other biofloc treatments, MG1 (11.56±1.34 g) and B1 (12.53±1 g). Probiotic mixes were found to further improve biofloc performance [31]. Similar findings were reported on the survival and growth rate of shrimps in biofloc systems using mixed probiotics [32]. This might be due to the consumption of microbial floc as an alternative natural feed for cultured fish. Biofloc contains up to 30–53% crude proteins [24,33] that improve feed efficiency. Both commercial pellet and microbial flocs (Phytoplankton, zooplankton, diatoms, fungi and microbes) are consumed by the cultured species in the probiotic-based biofloc system, which therefore show significant growth performance improvements of the cultured species [34,35,36,37,38]. In initial studies, the probiotics used had a beneficial effect on the growth performance, water quality, and disease resistance of cultured fish. Probiotic *L. fusiformis* caused the improved growth and health status of pacific white shrimp, *Penaeus vannamei* [39]. Similarly, the probiotic *B.amyloliquefaciens* was able to enhance nutrient metabolism and reduce oxidative stress and pathogen resistance in zebrafish, *Danio rerio* [40], and Nile tilapia, *Oreochromis niloticus* [41]. Furthermore, *E.hirae* was shown to enhance immunity through immune modulators in hybrid catfish (*Clariasgariepinus × Clarias macrocephalus*) [42].

### 4.3. Water Quality

In this experiment, the BFT water quality parameters were within the optimum range for red hybrid tilapia culture [43]. The nitrogen concentration (NO_2_-N and NH_4_-N) was significantly (*p* < 0.05) lower in all the biofloc groups compared to the control. By contrast, the higher level of NO_3_-N in the biofloc groups indicated the presence of nitrifying bacteria converting the NH_4_-N to NO_2_-N and then to NO_3_-N [20,44]. It was reported that NO_3_-N exhibited a low toxicity and was tolerable to cultured species [45,46,47] compared to NO_2_-N and NH_4_-N. It was suggested to perform water exchanges to maintain sufficient water quality.

### 4.4. Bacterial Challenge and Histopathology

The current study showed that red hybrid tilapia fingerlings cultured in biofloc groups with mixed probiotics (S4) demonstrated the highest survival, recorded at 83 ± 1.43%, showing higher resistance against *S. agalactiae* infection when compared to S1 (control group with *S. agalactiae*), with only a 40 ± 0.34% survival rate. In addition, other biofloc groups (S2 and S3) also showed better survival after being challenged with *S. agalactiae*. The increased resistance of the fingerlings in biofloc tanks might have been due to the optimal nutrients obtained in the biofloc. Biofloc have been reported to provide additional essential nutrients, including proteins, lipids, essential fatty acids, minerals, vitamins, and exogenous digestive enzymes [48,49] which might improve the growth performance and nutritional status of fish.

The higher survival in group S4 (biofloc with MP) indicated the higher resistance of the tilapia in the group against *S. agalactiae* infection, which was correlated with MP’s ability to inhibit pathogen growth in the in vitro assay. The lower *S. agalactiae* infection rate in the biofloc system might also have been due to the microbial balance, which enhanced the growth rate and improved the disease resistance of the fish [48,49]. Several clinical presentations of *S. agalactiae* infection in tilapia were observed, including erratic swimming and corneal opacity, and fin and internal organ haemorrhage. The clinical signs were similar to those seen in red hybrid tilapia challenged with *S. agalactiae* by Alsaid et al. [11] and Amal et al. [50]. The clinical symptoms caused by any pathogen, according to Yiagnisis and Athanassopoulou [51], were dependent on a number of factors, including the type of host, the age of the organism, and the stage of illness, whether acute or chronic.

Histopathological examination provides a methodological platform to determine the effects of bacteria in various organs. The gills of healthy fish, such as the control, showed a neat order of primary and secondary lamellae that could be readily distinguished. As seen in the S1 group, damaged gills with congestion could be identified by blood accumulation that caused swelling. The congestion was caused by a reduction in the venous blood flow, which was a passive process (increased blood volume in a vessel located in gill lamellae). This was often linked to stress, which caused cellular necrosis. As a result, erythrocyte congestion was common in the marginal channel (telangiectasia) [52]. Based on the liver histology of the selected groups, S4 showed similar tendencies to the control. As compared to the control, the histopathological changes observed in the livers of the group S1 fish revealed marked degeneration of the hepatocytes and congestion in the sinusoid due to the inability of the liver to detoxify the foreign body, resulting in liver dysfunction and, eventually, death [53]. *Streptococcus agalactiae* infection in the red hybrid tilapia livers demonstrated the vacuolation of hepatocytes, with fatty changes in response to the reduced blood flow due to congestion of the blood vessels and thrombosis of the portal blood vessels [54]. Histological analysis of the kidney in this study found that the S1 groups showed severe haemorrhage and thrombosis accompanied by infiltration of inflammatory cells similar to the experimental infection in *Oreochromis* sp. with *S. agalactiae* [55]. In addition, the control and S4 showed glomerulus vacuolation. Necrotic cells feature diminishing nuclei (pyknosis) and karyolysis that expand, blur or disappear. According to Woo and Buchman [56], necrosis is a condition in which tissue activity decreases and is characterized by the slow loss of certain cells from a tissue. As a result, cell death occurs shortly afterwards. In addition, the probiotics added in the bioflocsystem were able to enhance bacterial diversity in the ecosystem and bacterial abundance in the larval intestinal tract affected by the surrounding environment through feed intake [57]. This indicated that the addition of selected probiotics into the biofloc system improved its productivity and disease resistance. However, further studies should be performed on the microbial abundance in the culture system, which might improve the effectiveness of MP in the biofloc system.

## 5. Conclusions

In this study, the selected mixed probiotics that consisted of the *L. fusiformis* strain SPS11, the *B. amyloliquefaciens* strain L9, and the *E. hirae* strain LAB3 showed positive inhibition towards *S. agalactiae* and *S. iniae* through in vitro assay. In the in vivo assay, the results indicated the ability of this mix to improve the growth performance of red hybrid tilapia. The nitrogen concentration (NO_2_-N and NH_4_-N) was significantly lower in all the biofloc groups, which meant that the water quality was not affected by the high bacterial load. A significantly higher survival rate was observed in the biofloc group with the addition of probiotics after being challenged by *S. agalactiae*. Therefore, it could be concluded that the addition of probiotics demonstrated beneficial effects on the water quality, growth performance, and disease resistance of red hybrid tilapia in the biofloc system. The application of mixed probiotics in the biofloc systems could reduce the overall chances of pathogenic outbreaks and further enhance the efficacy of the system.

## Figures and Tables

**Table 1:** Experimental groups of probiotic addition in tilapia biofloc culture.

Code	Group
C	Control: Freshwater
B1	Control: Biofloc
MG1	Biofloc with commercial probiotics MG1
MP	Biofloc with MP (*Lysinibacillus fusiformis* SPS11, *Enterococcus hirae* LAB3, *Bacillus amyloliquefaciens* L9)

**Table 2:** Test groups of tilapia cultured in freshwater and biofloc group against pathogenic *Streptococcus agalactiae* in in vivo challenge assay.

Code	Group
CS	Control: Freshwater only
S1	*Streptococcus agalactiae* 10^8^ CFU mL^−1^
S2	Biofloc + *Streptococcus agalactiae* 10^8^ CFU mL^−1^
S3	Biofloc + MG1 + *Streptococcus agalactiae* 10^8^ CFU mL^−1^
S4	Biofloc + MP + *Streptococcus agalactiae* 10^8^ CFU mL^−1^

**Table 3:** Mean ± SEM of inhibition zones in mm of different probiotics against *Streptococcus agalactiae* and *Streptococcus iniae* at two different concentrations.

	Pathogen	*S. agalactiae*		*S. iniae*	
Probiotic		10^8^ CFU mL^−1^	10^6^ CFU mL^−1^	10^8^ CFU mL^−1^	10^6^ CFU mL^−1^
SPS11		7.6 ± 0.4 ^a^	5.4 ± 0.4 ^b^	6.65 ± 0.35 ^a^	4.7 ± 0.7 ^b^
L9		12.6 ± 0.6 ^a^	8.8 ± 0.2 ^b^	11.7 ± 0.7 ^a^	9.4 ± 0.4 ^b^
LAB 3		7.75 ± 0.75 ^a^	5.6 ± 0.4 ^c^	6.7 ± 0.7 ^b^	5.7 ± 0.3 ^c^
MP		10.2 ± 0.2 ^a^	8.05 ± 0.95 ^c^	10.75 ± 1 ^a^	9.3 ± 0.7 ^b^
MG1		4.65 ± 0.4 ^a^	2.25 ± 0.25 ^c^	3.4 ± 0.6 ^b^	1.7 ± 0.3 ^c^

SPS11: *Lysinibacillus fusiformis,* L9: *Bacillus amyloliquefaciens*, LAB3: *Enterococcus hirae*, MP: Mixed probiotics (SPS11, L9, and LAB3), MG1: Commercial probiotics. Supercript ^a,b,c^ at different alphabetical letters indicate a statistically significant difference (One-way ANOVA, *p* < 0.05).

**Table 4:** Mean values ± SEM of the growth parameters of red hybrid tilapia fingerling cultured in biofloc system with addition of probiotics.

Parameters	CS	B1	MG1	MP
Survival (%)	91 ± 0.91 ^a^	84 ± 1.78 ^a^	89 ± 1.28 ^a^	90 ± 1.16 ^a^
IBW (g)	0.57 ± 0.05 ^a^	0.53 ± 0.02 ^a^	0.4 ± 0.06 ^a^	0.51 ± 0.05 ^a^
FBW (g)	8.051 ± 1.4 ^c^	12.53 ± 1 ^b^	11.56 ± 1.34 ^b^	15.07 ± 1.30 ^a^
SGR (% day^−1^)	2.05 ± 0.20 ^a^	3.07 ± 0.33 ^b^	2.83 ± 0.30 ^b^	3.73 ± 0.23 ^b^
FCR	1.39 ± 0.08 ^a^	0.92 ± 0.10 ^b^	1.0 ± 0.10 ^b^	0.76 ± 0.04 ^b^

IBW = Initial body weight, FBW = Final body weight, SGR = Specific growth rate, FCR = Food conversion rate, B1 = Biofloc control (no probiotics), MG1 = Biofloc with MG1, MP = Biofloc with mixed probiotic. Supercript ^a,b,c^ at different alphabetical letters indicate a statistically significant difference (One-way ANOVA, *p* < 0.05).

**Table 5:** Mean ± SEM of water quality parameters during the culture of red hybrid tilapia fingerling in biofloc system with probiotics.

Parameters	CS	B1	MG1	MP
Temperature (°C)	27.57 ± 0.5 ^a^	27.65 ± 0.1 ^a^	27.48 ± 0.14 ^a^	27.61± 0.08 ^a^
DO (mg L^−1^)	3.53 ± 0.35 ^a^	3.24 ± 0.23 ^a^	3.45 ± 0.34 ^a^	3.40 ± 0.35 ^a^
pH	6.32 ± 0.08 ^a^	6.50 ± 0.01 ^a^	6.67± 0.03 ^a^	6.51± 0.02 ^a^
NO_2_-N (mg L^−1^)	2 ± 0.14 ^a^	1.45 ± 0.21 ^b^	1.32 ± 0.08 ^b^	0.84 ± 0.07 ^c^
NO_3_-N (mg L^−1^)	0.8 ± 0.31 ^c^	2.20 ± 0.45 ^b^	2.70 ± 0.3 ^a^	2.00 ± 0.22 ^b^
NH_4_-N (mg L^−1^)	3.8 ± 0.3 ^a^	2.97 ± 0.17 ^b^	3.20 ± 0.29 ^b^	2.60 ± 0.08 ^c^
SD (mg L^−1^)	-	23.00 ± 2.3 ^b^	27.30 ± 2.7 ^b^	31.27 ± 3.1 ^a^

DO = Dissolved oxygen, NO_2_-N = Nitrite, NO_3_-N = Nitrate, NH_4_-N = Ammonium, SD = Settled biomass, B1 = Biofloc control, MG1 = Biofloc with MG1, MP = Biofloc with mixed probiotic. Supercript ^a,b,c^ at different alphabetical letters indicate a statistically significant difference (One-way ANOVA, *p* < 0.05).

## Data Availability

All data generated or analyzed during this study have been included in this article.
